# Analysis of influencing factors of AIDS epidemic in Kunming based on PCA-GWR method

**DOI:** 10.3389/fpubh.2025.1658700

**Published:** 2025-12-10

**Authors:** LiangTing Zheng, Bin Liao, Yi Li, Jun Lian, Jingying Wang, Yanli Ma, Ruilin Feng, Wenying Hu, Xianfu Bai

**Affiliations:** 1 Yunnan Earthquake Agency, Kunming, China; 2 Faculty of Geography, Yunnan Normal University, Kunming, China; 3 AIDS/STD Control and Prevention, Kunming Center for Disease Control and Prevention, Kunming, China

**Keywords:** HIV/AIDS, geographically weighted regression, principal component analysis, influencing factor, Kunming city

## Abstract

**Background:**

As of 2024, an estimated 40.8 million people worldwide were living with HIV, making HIV/AIDS one of the most pressing global public health challenges. Accurate identification of the factors shaping the HIV/AIDS epidemic is essential for developing targeted prevention and control strategies.

**Methods:**

This study uses Principal Component Analysis (PCA) and Geographically Weighted Regression (GWR) to examine spatially varying associations between HIV/AIDS prevalence and three domains—socioeconomic conditions, educational attainment, and healthcare capacity—using Kunming, China, as a case study.

**Results:**

The results indicate that: (1) the effects of socioeconomic conditions, educational attainment, and healthcare capacity on HIV/AIDS prevalence exhibit significant spatial heterogeneity across Kunming; (2) in the northern part of Kunming—particularly Dongchuan District, Luquan County, Xundian County, and Fumin County—higher prevalence is largely associated with the combined influence of lower economic development and limited educational attainment, with economic development negatively correlated with prevalence and lower educational levels positively correlated with infection rates; and (3) HIV/AIDS prevalence is also related to the level of healthcare services, which is generally negatively correlated with prevalence—i.e., better healthcare conditions are associated with lower infection rates—although areas with more advanced healthcare systems may show higher detection and reporting.

**Conclusion:**

The AIDS epidemic results from the interplay of multiple factors, with dominant determinants varying geographically. These findings provide spatially explicit evidence to guide targeted policy development and resource allocation.

## 1 Introduction

According to global HIV/AIDS data released by the Joint United Nations Programme on HIV/AIDS (UNAIDS) in 2024, an estimated 40.8 million people worldwide were living with HIV, by the end of that year, with 1.3 million new infections and approximately 630,000 AIDS-related deaths reported (1). The high transmissibility of HIV and the substantial mortality associated with AIDS progression pose significant challenges to global public health systems. Yunnan Province, located in China’s southwestern border region, was among the earliest and most severely affected areas by indigenous HIV transmission (2). Its provincial capital, Kunming, has maintained persistently high HIV/AIDS prevalence, functioning both as a critical hub in regional transmission networks and as a focal point for innovation and demonstration in prevention and control. Identifying the key determinants underlying intra-urban disparities in HIV prevalence and generating spatially explicit evidence to inform targeted interventions remain critical questions that current HIV prevention research and governance urgently need to address.

Epidemiological studies in Yunnan have revealed marked regional disparities in the spatial distribution of HIV infection rates, and the widespread application of geographic information technologies in surveillance and analysis offers new opportunities to address spatial analytical challenges. Spatial analytical methods have become increasingly prevalent in HIV-related research. Tools such as spatial autocorrelation (e.g., Moran’s I) and hotspot detection (Getis-Ord Gi*) have been widely used to characterize spatial patterns and temporal dynamics of the HIV epidemic, enabling the identification of statistically significant high-risk clusters (3, 4). This approach effectively describes and analyzes spatial distribution characteristics, providing a scientific basis for identifying high-incidence areas and informing policy formulation (5).

To identify the factors underlying regional disparities in HIV/AIDS prevalence, spatial analysis should be integrated with an examination of influencing factors. Models such as ordinary least squares (OLS) regression and the geographical detector have been widely applied to explore how economic, educational, and healthcare factors are associated with HIV prevalence. For instance, Xie et al. used the geographical detector to analyze HIV incidence and socioeconomic factors in China from 2009 to 2019, finding that urbanization rate, per capita disposable income, and population density had strong explanatory power, whereas improved healthcare development tended to suppress the spatial spread of HIV (6). Maranhão et al. employed OLS regression to assess the influence of social determinants on HIV incidence in Piauí State, showing that socioeconomic status and educational attainment were the primary determinants (7). However, such global models are inadequate for capturing spatial heterogeneity, where model coefficients vary across space. Prior studies indicate that the drivers of HIV prevalence differ by location. In contrast, geographically weighted regression (GWR) incorporates geographic location into the regression framework, allowing direct observation of how the effects of explanatory variables vary spatially (8, 9). This method is particularly suitable for exploring the spatial mechanisms of “where” and “why” such variations occur. Wang et al. conducted a comparative analysis of OLS and GWR, demonstrating GWR’s superior capability in identifying factors shaping HIV/AIDS prevalence patterns (10).

When analyzing influencing factors, multicollinearity among variables is likely; thus dimensionality reduction should be performed beforehand to improve robustness. In this study, the dimensionality reduction concept of principal component analysis (PCA) was applied to transform multiple correlated influencing factors into several uncorrelated composite indicators. This approach not only preserves as much information as possible from the original variables but also simplifies the data structure (11).

In summary, existing research still lacks street-level spatial analyses of the HIV/AIDS epidemic that comprehensively account for variation in socioeconomic factors. Therefore, this study integrates PCA and GWR to identify the spatially heterogeneous effects of economic, educational, and healthcare factors on the HIV/AIDS epidemic, providing a new perspective on the multidimensional social determinants of the disease.

## 2 Study area and data

### 2.1 Overview of the study area

**Figure 1:**
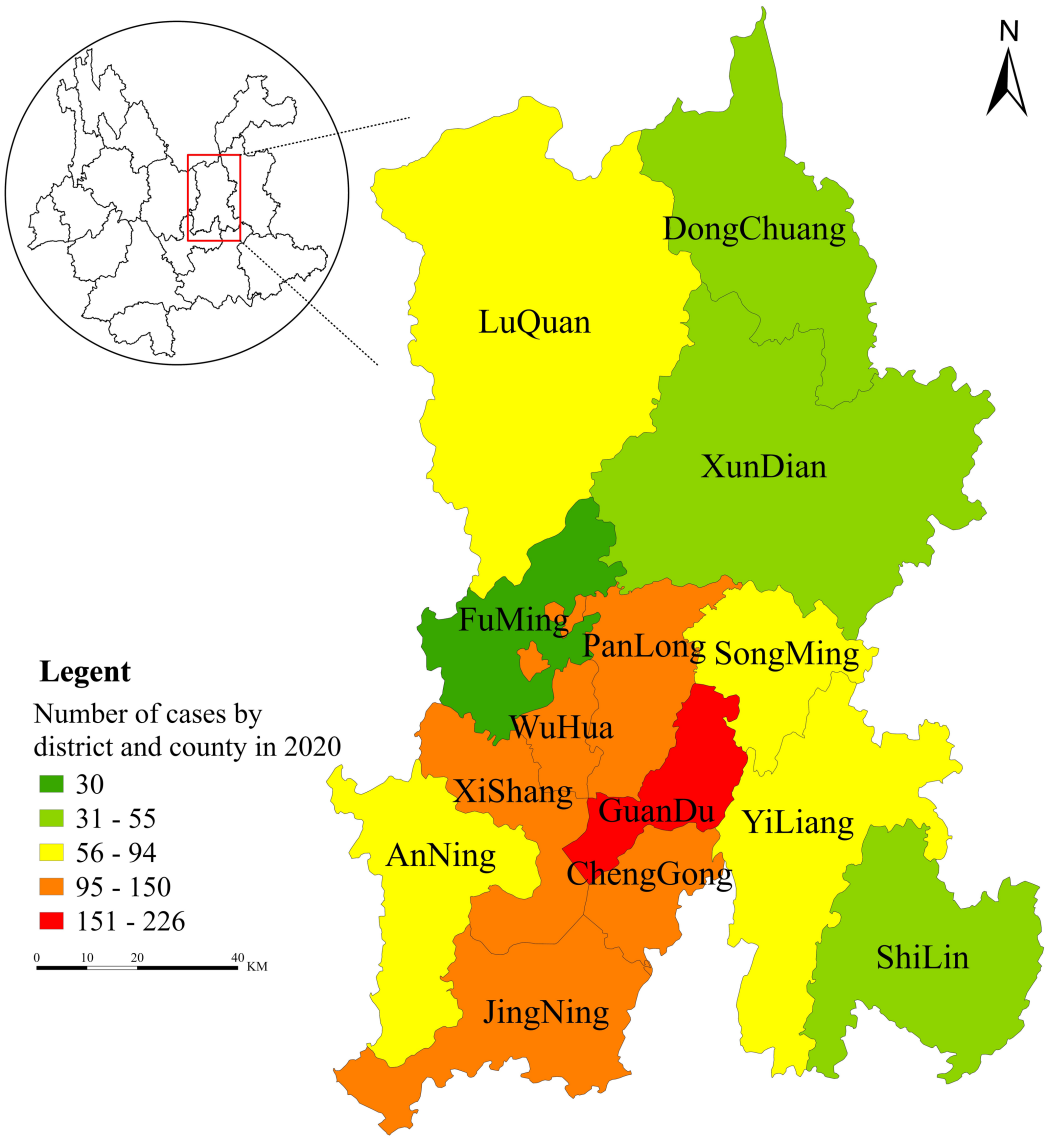
Spatial distribution of the number of AIDS cases in districts (counties) of Kunming in 2020.

Kunming, the capital of Yunnan Province, lies between 102°10′–103°40′E and 24°23′–26°22′N. Administratively, Kunming comprises seven municipal districts, three counties, one county-level city, and three autonomous counties; its resident population was 8.46 million in the 2020 census (12). In 2020, Kunming reported 1,453 new HIV cases and 54 AIDS-related deaths. During data preprocessing, we excluded decedents and non-mainland Chinese cases (foreign nationals and residents of Hong Kong, Macao, and Taiwan). The analytical dataset included laboratory-confirmed cases with validated case report forms. Figure 1 shows the spatial distribution of HIV/AIDS case counts across district- and county-level administrative units in Kunming in 2020.

### 2.2 Data collection and processing

#### 2.2.1 Basic data collection and processing

Based on data from the National AIDS Information System, we constructed the Kunming HIV/AIDS epidemiologic database by overlaying local population statistics and administrative boundary data.

1. Kunming’s 2020 HIV/AIDS case data were obtained from the National Integrated HIV/AIDS Prevention and Control Information Management System (China). After data screening, case records were standardized and geocoded from residential addresses; records lacking verifiable addresses were excluded. Geocoding was performed with a map-location tool to convert addresses to geographic coordinates (latitude/longitude), thereby establishing a geospatial database of HIV cases in Kunming. To protect patient privacy, all personally identifiable information was anonymized prior to statistical analysis.
2. Administrative boundary data for Kunming were obtained from the National Geographic Information Resource Catalog Service System of the Ministry of Natural Resources (https://www.webmap.cn; accessed 15 October 2021). According to the most recent administrative division data, Kunming Municipality comprises 14 county-level units (districts and counties) and 139 township-level units (subdistricts and towns).
3. Population statistics for Kunming at the district/county and subdistrict (street) levels were obtained from the Seventh National Population Census (2020) conducted by the National Bureau of Statistics of China. We constructed a geospatial database by joining district/county- and subdistrict/township-level demographic data with Kunming’s administrative boundary vector datasets.

#### 2.2.2 Data collection and processing of factors influencing the prevalence of HIV/AIDS

High-risk behaviors—such as unprotected sex, multiple concurrent sexual partnerships, and injection drug use—are primary social drivers of HIV/AIDS transmission, and these behaviors are influenced by socioeconomic conditions, educational attainment, and individual characteristics (13, 14). Furthermore, the level of healthcare services substantially influences HIV/AIDS case detection and awareness. Guided by the epidemiological characteristics of HIV/AIDS in Kunming and relevant literature, we selected measurable indicators across four domains—economic development, social progress, educational attainment, and healthcare capacity—to analyze the factors influencing disease prevalence.

1. The annual GDP raster data for Kunming were obtained from the Resource and Environmental Science and Data Center of the Chinese Academy of Sciences (1 km spatial resolution). District- and county-level annual GDP values were then aggregated using a subdistrict-level zonal statistics tool.
2. Road network data for Kunming were sourced from the OpenStreetMap (OSM) road dataset. After preprocessing, road network density was calculated for each district and county in Kunming.
3. Leisure and entertainment points of interest (POIs) in Kunming were obtained from the Gaode Map (Amap) API, accessed in September 2021. The dataset includes categories such as hotels, spas, bars, and KTVs. Records with incomplete address information were excluded, yielding a final analytical sample of 15,838 valid entries. POI coordinates were geocoded using the map-location tool, followed by kernel density estimation (KDE) at the district/county level.
4. All indicators—including urban and rural residents’ disposable income, urbanization rate, unemployment rate, the share of the population aged 15–59 years, illiteracy rate, counts of individuals with tertiary education (junior college and above), upper secondary education (including vocational secondary), lower secondary education, and primary education, as well as the numbers of hospital beds and health technicians—were obtained from the Kunming Statistical Bulletin on National Economic and Social Development (2020). These data were compiled at the district/county level; using district/county centroids as interpolation points, we performed spatial interpolation to generate estimates at the street (subdistrict) level.

Across four domains—economic conditions, social development, educational attainment, and healthcare—we selected 19 candidate determinants of the HIV/AIDS epidemic in Kunming and constructed the corresponding indicator layers, as summarized in Table 1. We then integrated the Kunming HIV/AIDS epidemiologic database with the influencing-factor indicator system to enable spatial analysis of prevalence determinants. To remove scale effects, improve analyzability, and enhance model generalizability, all variables were standardized prior to analysis.

**Table 1:** Indicator system of influencing factors of HIV/AIDS prevalence in Kunming.

Variables	Indicators	Unit
Economic life	Annual GDP	100 million yuan
	Disposable income of rural residents	Yuan
	Disposable income of urban residents	Yuan
	Road density	Km/km^2^
	Leisure and entertainment POI	Piece
Social development	Population density	Person/km^2^
	Urbanization rate	%
	Proportion of population aged 15–59	%
	Urban registered unemployment rate at the end of the year	%
Educational attainment	Illiteracy rate	%
	Nine-year compulsory education completion rates	%
	Number of persons with university (college and above) education	Person
	Number of persons with high school (including secondary school) education	Person
	Number of persons with lower secondary education	Person
	Number of persons with elementary school education	Person
Healthcare	Number of beds in health facilities	Piece
	Number of healthcare technicians	Person
	VCT spatial accessibility	–
	PITC spatial accessibility	–

## 3 Methods

### 3.1 Principal component analysis

In regression analyses, substantial intercorrelations among predictors can induce multicollinearity. Accordingly, prior to model estimation, we applied principal component analysis (PCA) to mitigate this issue and subsequently incorporated the resulting orthogonal components into geographically weighted regression (GWR) to quantify their spatially varying effects.

Principal component analysis (PCA) reduces a set of variables to a small number of composite factors—principal components—that retain as much information as possible from the original variables (11). The PCA framework is mathematically defined as follows: Consider n spatial units (street-level divisions), each characterized by p AIDS prevalence influence factors $\left(X_{1},X_{2},\cdots X_{p}\;\right)$ , which collectively form an n×p dimensional matrix. As shown in Equation 1.


$$X=[\begin{array}{c} x_{11}x_{12}\cdots x_{1p}\\ x_{21}x_{22}\cdots x_{2p}\\ \cdots \\ x_{n1}x_{n2}\cdots x_{\mathrm{np}} \end{array}]=(X_{1},X_{2},\cdots ,X_{P})$$ (1)

where $\left(X_{1},X_{2},\cdots X_{P}\right)$ are original HIV/AIDS prevalence indicators. As the different dimensions of the raw HIV/AIDS epidemic factors, when performing principal component extraction, the original factors are first standardized. The correlation coefficient matrix was computed for the standardized variables, from which the eigenvalues $\left(\lambda_{1},\lambda_{2},\cdots \lambda_{p}\right)$ and corresponding eigenvectors were derived. These eigenvalues were subsequently sorted in descending order $\left(\lambda_{1}>\lambda_{2}>\cdots \lambda_{p}\right)$ to determine the principal components. The eigenvector $F_{1}$ corresponding to the largest eigenvalue $\lambda_{1}$ constitutes the first principal component, while $F_{2}$ associated with $\lambda_{2}$ represents the second principal component, with subsequent components following analogously in descending order of their explained variance. $F_{1},F_{2},\cdots F_{P}$ are new variable indicators. As shown in Equation 2.


$$\left\{\begin{array}{c} F_{1}=a_{11}X_{1}+a_{12}X_{2}+\cdots +a_{1p}X_{P}\\ F_{2}=a_{21}X_{1}+a_{22}X_{2}+\cdots +a_{2p}X_{P}\\ \cdots \\ F_{P}=a_{p1}X_{1}+a_{p2}X_{2}+\cdots +a_{\mathrm{pp}}X_{P} \end{array}\right\}$$ (2)

The formula satisfies, $a_{1i}^{2}+a_{2i}^{2}\cdots +a_{\mathrm{ip}}^{2}=1$ ; $F_{i},F_{j}(i\neq j,i,j=1,2,\cdots ,p)$ , are uncorrelated and the variance of $\;F_{1},F_{2},\cdots ,F_{p}$ is gradually decreasing, i.e., $\mathrm{Var}(F_{1})>\mathrm{Var}(F_{2})>\cdots >\mathrm{Var}(F_{p})$ , the greater the variance of the principal components contains more information about the original variables.

The optimal number of principal components is determined by cumulative explained variance, with the common practice of retaining components that together account for 80–90% of the total variance in the original data (13). The formulas for the contribution rate and the cumulative contribution rate are provided in Equations 3 and 4.


$$\text{Contribution Rate}=\frac{\lambda_{i}}{\sum_{k=1}^{p}\lambda_{k}},i=1,2,\cdots ,p$$ (3)

$$\text{Cumulative Contribution Rate}=\frac{\sum_{k=1}^{i}\lambda_{k}}{\sum_{k=1}^{p}\lambda_{k}},i=1,2,\cdots ,p$$ (4)

where λ is the eigenvalue of each factor and k is the first k principal components.

Using PCA, the 19 selected factors were synthesized into new composite indicators, which were then incorporated into a geographically weighted regression (GWR) model to analyze determinants of HIV/AIDS prevalence in Kunming. This approach mitigates multicollinearity arising from numerous predictors while simplifying the set of influencing factors.

### 3.2 Geographically weighted regression model

The spread of HIV/AIDS is closely associated with economic conditions, population mobility, and healthcare provision. Differences in regional socioeconomic development and natural environments lead to spatial heterogeneity in the determinants of HIV/AIDS prevalence in Kunming. Geographically weighted regression (GWR), proposed by Fotheringham, is an extension of ordinary least squares (OLS). Based on the principle of local regression, it embeds the spatial locations of samples into the regression equation to enable location-specific parameter estimation and to quantify the effects of individual factors at different geographic locations (14). The expression is given in Equation 5.


$$x_{i}=\beta_{0}(u_{i},v_{i})+\sum_{m=1}^{k}\beta_{m}(u_{i},v_{i})F_{\mathrm{mi}}+\varepsilon_{i}$$ (5)

Where $x_{i}$ denote the HIV/AIDS prevalence rate in Kunming’s ith street; $\left(u_{i},v_{i}\right)$ is the geographical coordinate of the ith street; $\beta_{0}\;(u_{i},v_{i})\;$ is the regression constant of the i th street; k denotes the k th principal component extracted; $\beta_{m}\;(u_{i},v_{i})\;$ as the regression coefficient for the m th principal component at the i th street; $F_{\mathrm{mi}}$ as the value of the m th principal component of the i th street; $\varepsilon_{i}$ as the spatially distributed random error term of the i th street.

In the GWR model, regression coefficients vary with the geographic location of each observation, and the degree of influence is expressed by a distance-based kernel function (15). Common kernels include the distance-threshold, inverse-distance, Gaussian, and bisquare functions, among which the Gaussian kernel is widely used for its general applicability (8). The mathematical form of the Gaussian kernel is given in Equation 6.


$$w_{\mathrm{ij}}=\exp(-{\left(d_{\mathrm{ij}}/b\right)}^{2})$$ (6)

where $w_{\mathrm{ij}}$ is the weight of the data point; $d_{\mathrm{ij}}$ is the distance between data point i and regression j ; and b is the bandwidth. In GWR, two key choices are the kernel function and the optimal bandwidth. An excessively large bandwidth inflates regression parameter estimates, whereas an excessively small bandwidth deflates them. To avoid errors due to inappropriate bandwidths, we determine the optimal bandwidth using the Akaike Information Criterion (AIC), selecting the bandwidth that yields the minimum AIC value. Accordingly, this study employs a Gaussian kernel with AIC-based bandwidth selection and incorporates the newly extracted principal components into the GWR model to analyze the determinants of the HIV/AIDS epidemic in Kunming.

Compared with traditional OLS, GWR reveals spatial variation in relationships between variables across regions. Given the substantial disparities in economic, educational, and healthcare conditions within Kunming, a single global regression cannot accurately capture how these factors vary in influence across areas. Accordingly, we first applied principal component analysis (PCA) to reduce the dimensionality of 19 socioeconomic and healthcare variables and to extract independent composite factors, thereby minimizing multicollinearity. We then incorporated these components into a geographically weighted regression (GWR) to conduct spatially weighted analysis of HIV/AIDS prevalence and identify key determinants across regions. This combined PCA–GWR framework more effectively characterizes spatial heterogeneity and provides a scientific basis for region-specific HIV/AIDS prevention and control.

## 4 Results

### 4.1 Principal component analysis of factors influencing HIV/AIDS prevalence in Kunming

To evaluate the suitability of the selected determinants of HIV/AIDS prevalence for factor analysis, we conducted Kaiser–Meyer–Olkin (KMO) and Bartlett’s test of sphericity in SPSS. The results are reported in Table 2.

**Table 2:** KOM and Bartlett test results.

Variables	Value	Category
KMO measure of sampling adequacy		0.804
Bartlett’s test of sphericity	Approximate chi-square	4312.577
	Degrees of freedom	78
	*p*-value	0.000

As presented in Table 2, the KMO measure for the selected influencing factors is 0.804, suggesting adequate similarity in the strength of correlations among the variables and supporting the suitability of the data for factor analysis. Furthermore, Bartlett’s test of sphericity shows a significance level of 0.000, indicating that the null hypothesis of sphericity is rejected and significant correlations exist among the variables, thus verifying the appropriateness of applying factor analysis.

The principal components selected by PCA capture the key information contained in the influencing factors; however, variables that are strongly correlated with prevalence but account for only a small proportion within the principal components may be overlooked (13). To address this issue, we first conducted correlation analyses between each factor and disease prevalence before performing PCA, and only factors showing strong correlations with prevalence were retained for PCA. Pearson correlation coefficients were used to characterize the strength of association between influencing factors and prevalence, with the results reported in Table 3.

**Table 3:** Analysis of the degree of correlation between the potential influencing factors of HIV/AIDS and the prevalence of HIV/AIDS in Kunming.

Variables	Indicators	Pearson correlation coefficient
Economic life	Annual GDP	−0.289**
	Disposable income of rural residents	0.390**
	Disposable income of urban residents	0.364**
	Road density	−0.250**
	Leisure and entertainment POI	0.609**
Social development	Population density	−0.055
	Urbanization rate	0.419**
	Proportion of population aged 15–59	0.387**
	Urban registered unemployment rate at the end of the year	−0.090
Educational attainment	Illiteracy rate	−0.383**
	Nine-year compulsory education completion rates	0.145
	Number of persons with university (college and above) education	0.360**
	Number of persons with high school (including secondary school) education	0.361**
	Number of persons with lower secondary education	0.394**
	Number of persons with elementary school education	0.278**
Healthcare	Number of beds in health facilities	0.256**
	Number of healthcare technicians	0.269**
	VCT spatial accessibility	0.214*
	PITC spatial accessibility	0.049

*Indicates statistical significance at the 95% confidence level. **Indicates statistical significance at the 99% confidence level.

As shown in Table 3, no statistically significant associations (*p* > 0.05) were observed between street-level HIV/AIDS prevalence in Kunming and (a) the annual registered unemployment rate, (b) the completion rate of nine-year compulsory education, (c) PITC spatial accessibility, or (d) population density. To ensure the robustness of the analysis, these four factors were excluded, and the remaining 15 factors that exhibited significant correlations with prevalence were retained for PCA. The explained variance of the retained components is reported in Table 4.

**Table 4:** Statistics of total variance explained by PCA of influencing factors of HIV/AIDS prevalence in Kunming in 2020.

Component	Initial eigenvalue			Extract the sum of squared loads	Contribution rate	Cumulative contribution rate
	Total	Contribution rate	Cumulative contribution rate	Total		
1	9.274	61.829	61.829	9.274	61.829	61.829
2	2.544	16.958	78.786	2.544	16.958	78.786
3	1.189	7.928	86.715	1.189	7.928	86.715
4	0.733	4.884	91.599			
5	0.567	3.778	95.377			
6	0.390	2.600	97.977			
7	0.149	0.995	98.972			
8	0.059	0.391	99.362			
9	0.038	0.257	99.619			
10	0.022	0.147	99.766			
11	0.019	0.130	99.896			
12	0.011	0.076	99.972			
13	0.003	0.018	99.990			
14	0.001	0.009	100.000			
15	0.000064	0.000	100.000			

As shown in Table 4, the first, second, and third principal components account for 61.829, 16.958, and 7.928% of the total variance, respectively, yielding a cumulative variance explained of 86.715%. Thus, the first three components capture 86.715% of the information in the original variables. Accordingly, we extracted these three components, transforming the 15 correlated HIV/AIDS prevalence variables into three mutually uncorrelated (orthogonal) components.

As summarized in Table 5, the characteristic variables corresponding to the three principal components are as follows. The first principal component (PC1) is dominated by four education-related variables—population with tertiary education (college degree or above), population with upper secondary education (including vocational school), illiteracy rate, and population with lower secondary education—with factor loadings all exceeding 0.90; we therefore label PC1 as education level. The second principal component (PC2) is characterized by annual GDP and road network density, both with loadings greater than 0.80; because the remaining variables exhibit substantially lower loadings (<0.80), PC2 is interpreted as economic development. The third principal component (PC3) is dominated by the spatial accessibility of voluntary counseling and testing (VCT) services, which shows the highest loading; accordingly, PC3 is labeled healthcare.

**Table 5:** Characterization variables of the new principal components.

Indicators	First principal component	Second principal component	Third principal component
Urbanization rate	0.976	−0.077	0.046
Number of persons with university (college and above) education	0.974	0.110	−0.006
Number of persons with high school (including secondary school) education	0.961	0.215	−0.085
Illiteracy rate	−0.946	0.175	−0.204
Disposable income of rural residents	0.925	−0.256	0.209
Number of persons with lower secondary education	0.923	0.225	−0.139
Disposable income of urban residents	0.903	−0.245	0.246
Number of healthcare technicians	0.882	0.264	−0.064
Number of beds in health facilities	0.848	0.286	−0.107
Proportion of population aged 15–59	0.794	−0.357	0.195
Number of persons with elementary school education	0.679	0.483	−0.344
Leisure and entertainment POI	0.598	0.007	−0.334
Annual GDP	−0.199	0.853	0.436
Road density	−0.072	0.840	0.504
VCT spatial accessibility	0.207	−0.572	0.536

### 4.2 Analysis of influencing factors of AIDS prevalence rate based on PCA-GWR

The three principal components derived from PCA were incorporated into the GWR model to estimate local regression coefficients. We then performed street-level spatial interpolation to visualize the 2020 spatial distribution of determinants of HIV/AIDS prevalence in Kunming.

#### 4.2.1 Impact analysis of the first principal component on the prevalence of AIDS in Kunming

**Figure 2:**
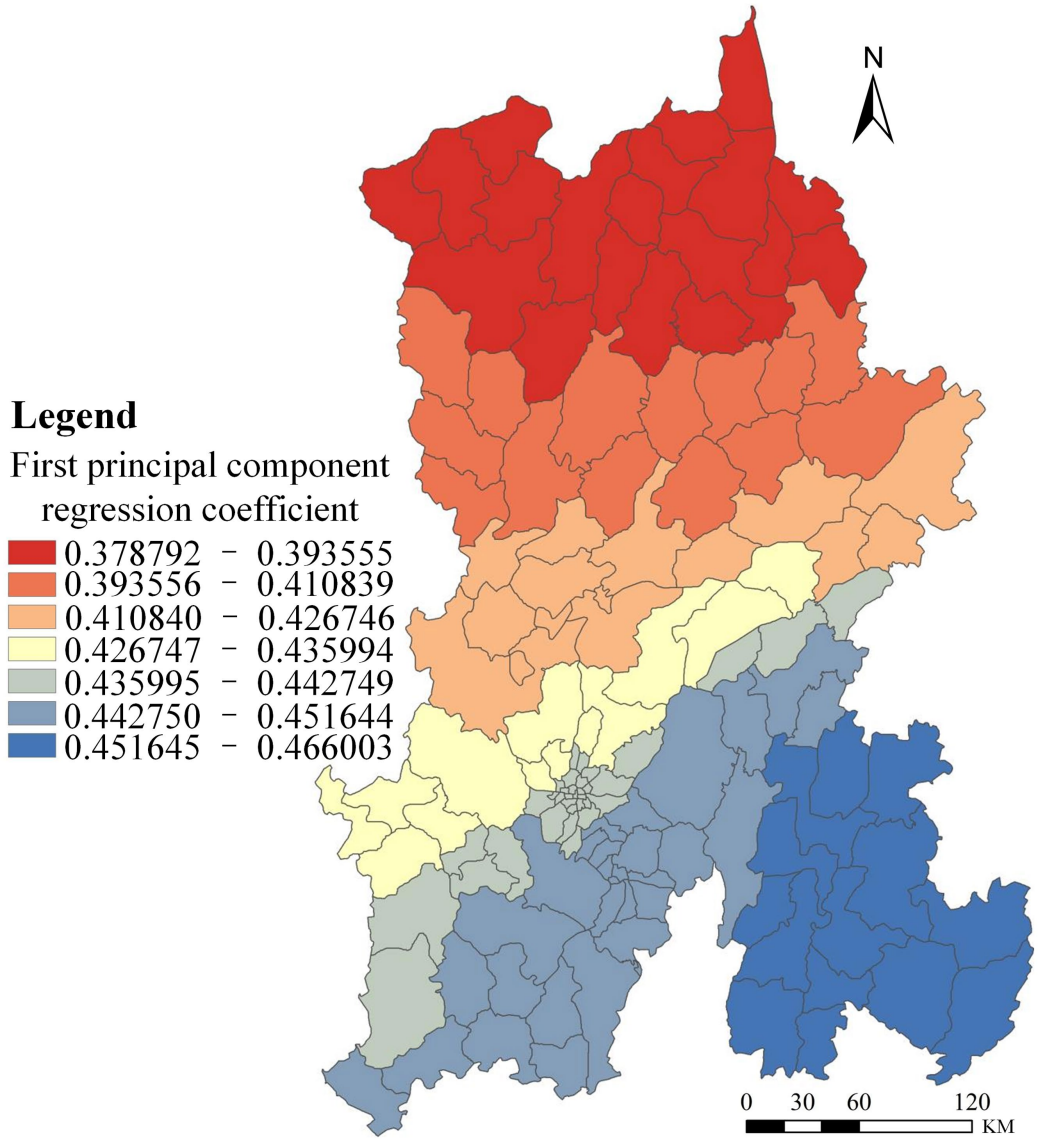
Spatial distribution of regression coefficients for the first principal component PCA-GWR model.

The first principal component primarily represents overall educational attainment in Kunming. As shown in Table 5, the loading for the population with tertiary education is high (0.974); likewise, the positive loadings for the populations with upper secondary education (0.961) and lower secondary education (0.923), as well as the negative loading for the illiteracy rate (−0.946), are also substantial. This indicates that the first principal component reflects differences in education level and, in essence, captures an educational gradient, with greater weights on lower-education indicators. The analysis of how Kunming’s overall educational attainment affects HIV/AIDS prevalence is presented in Figure 2. The model’s regression coefficients are all positive, implying a positive association between lower educational attainment and HIV/AIDS prevalence in Kunming; that is, areas with higher illiteracy rates and larger proportions of populations with lower education exhibit higher HIV/AIDS prevalence.

The effect of educational attainment on HIV/AIDS prevalence in Kunming shows a north-to-south decreasing gradient, with the strongest effects concentrated in the north-central region—particularly Dongchuan District, Luquan Yi and Miao Autonomous County, and Xundian Hui and Yi Autonomous County. Educational attainment shapes awareness and prevention capacity: populations with upper-secondary or lower education exhibit markedly lower HIV/AIDS knowledge and weaker self-protection, making them more vulnerable to infection and onward transmission. Illiteracy rates by district and county are reported in Table 6.

**Table 6:** Illiteracy rate statistics of Kunming districts and counties in 2020.

District/County	Illiteracy rate* (%)
Xundian Hui and Yi Autonomous County	6.73
DongChuan District	6.11
LuQuan Yi and Miao Autonomous County	5.09
Shilin Yi Autonomous County	3.88
JinNing District	3.86
Fumin County	3.84
YiLiang County	3.57
SongMing County	2.9
AnNing City	2.77
ChengGong District	1.76
GuanDu District	1.52
PanLong District	1.43
WuHua District	1.29
XiShan District	1.29

*The illiteracy rate were extracted from the 2020 Kunming Statistical Bulletin on National Economic and Social Development.

As shown in Table 5, the highest illiteracy rates are found in the northern Xundian Hui and Yi Autonomous County (6.73%), Dongchuan District (6.11%), and Luquan Yi and Miao Autonomous County (5.09%). Ethnic minority groups demonstrate more liberal attitudes toward sexuality and more susceptible to HIV than compared to the Han Chinese population. These three districts exhibit the strongest association between educational attainment and AIDS prevalence, driven primarily by limited HIV/AIDS knowledge, lower risk awareness, and greater engagement in high-risk behaviors. HIV awareness in the general population varies substantially by education level, with markedly higher awareness among individuals with tertiary education than among those with only primary or secondary schooling. In summary, in northern Kunming—Xundian County, Dongchuan District, Luquan County, Songming County, and Fumin County—lower educational attainment, weaker self-protective practices, and limited awareness of HIV prevention and care are key contributors to the regional burden of HIV/AIDS.

#### 4.2.2 Impact analysis of the second principal component on the prevalence of AIDS in Kunming

**Figure 3:**
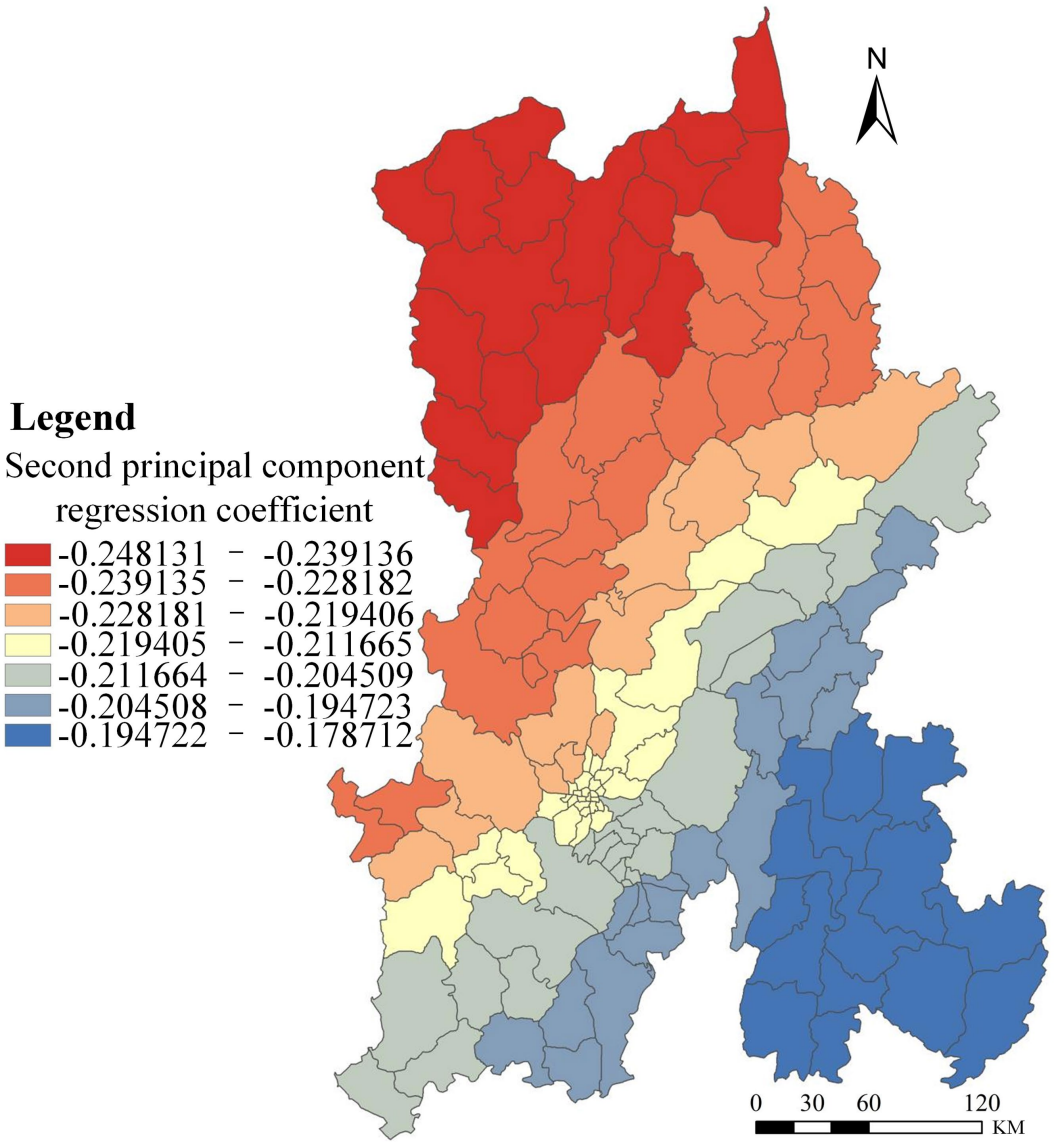
Spatial distribution of regression coefficients for the second principal component PCA-GWR model.

The second principal component primarily reflects economic conditions in Kunming. Its effect on the HIV/AIDS epidemic was analyzed, and the results are shown in Figure 3.

The effect of economic factors on HIV/AIDS prevalence in Kunming shows a north-to-south decreasing gradient, with regression coefficients ranging from −0.248131 to −0.17812 (all negative). These results indicate a negative association between economic development and HIV/AIDS prevalence: areas with lower economic development tend to have higher prevalence. In economically disadvantaged regions, livelihood pressures may prompt young adults to migrate for work, potentially increasing exposure to high-risk behaviors, including sex work and other unsafe practices. Among people who inject drugs (PWID), cost-saving behaviors—such as syringe sharing and use of non-sterile equipment—substantially elevate HIV transmission risk. Conversely, regions with higher economic development typically exhibit lower HIV/AIDS incidence, attributable to better access to healthcare resources and greater uptake of preventive measures (e.g., routine medical screening). Annual GDP statistics for Kunming’s districts and counties in 2020 are reported in Table 7.

**Table 7:** Annual GDP statistics of Kunming districts and counties in 2020.

District/County	Annual GDP (100 million yuan)
Xundian Hui and Yi Autonomous County	27,918
LuQuan Yi and Miao Autonomous County	31,929
DongChuan District	40,286
SongMing County	42,023
Shilin Yi Autonomous County	43,540
YiLiang County	45,938
JinNing District	59,742
Fumin County	63,077
AnNing City	93,853
PanLong District	105,362
XiShan District	108,043
ChengGong District	131,672
WuHua District	135,877
GuanDu District	145,011

As shown in Table 7, annual GDP in north-central Kunming—Xundian County, Luquan County, Dongchuan District, and Songming County—is comparatively low. Consistent with the effects of the first principal component, overall educational attainment in these areas is also relatively low. The combination of underdeveloped economic conditions and limited education constrains access to health education and is associated with low awareness of disease prevention; in some cases, pursuit of additional income may increase engagement in high-risk behaviors. By contrast, southern Kunming—Guandu, Wuhua, Chenggong, Xishan, and Panlong Districts—exhibits substantially higher annual GDP. Economic development in these districts coincides with improvements in related social conditions, including better healthcare access and stronger health consciousness and self-protective practices, which help reduce infection risk.

Notably, although Kunming’s overall economic level is negatively associated with HIV/AIDS prevalence, two indicators in Table 3—rural residents’ disposable income (0.390) and urban residents’ disposable income (0.364)—show positive correlations with prevalence. In conjunction with the PC1-based analysis, this suggests that in areas with lower educational attainment—where health awareness is weaker, labor out-migration is substantial, and rising incomes are not effectively converted into investments in health protection—growth in residents’ disposable income is positively associated with disease prevalence.

#### 4.2.3 Impact analysis of the third principal component on the prevalence of AIDS in Kunming

**Figure 4:**
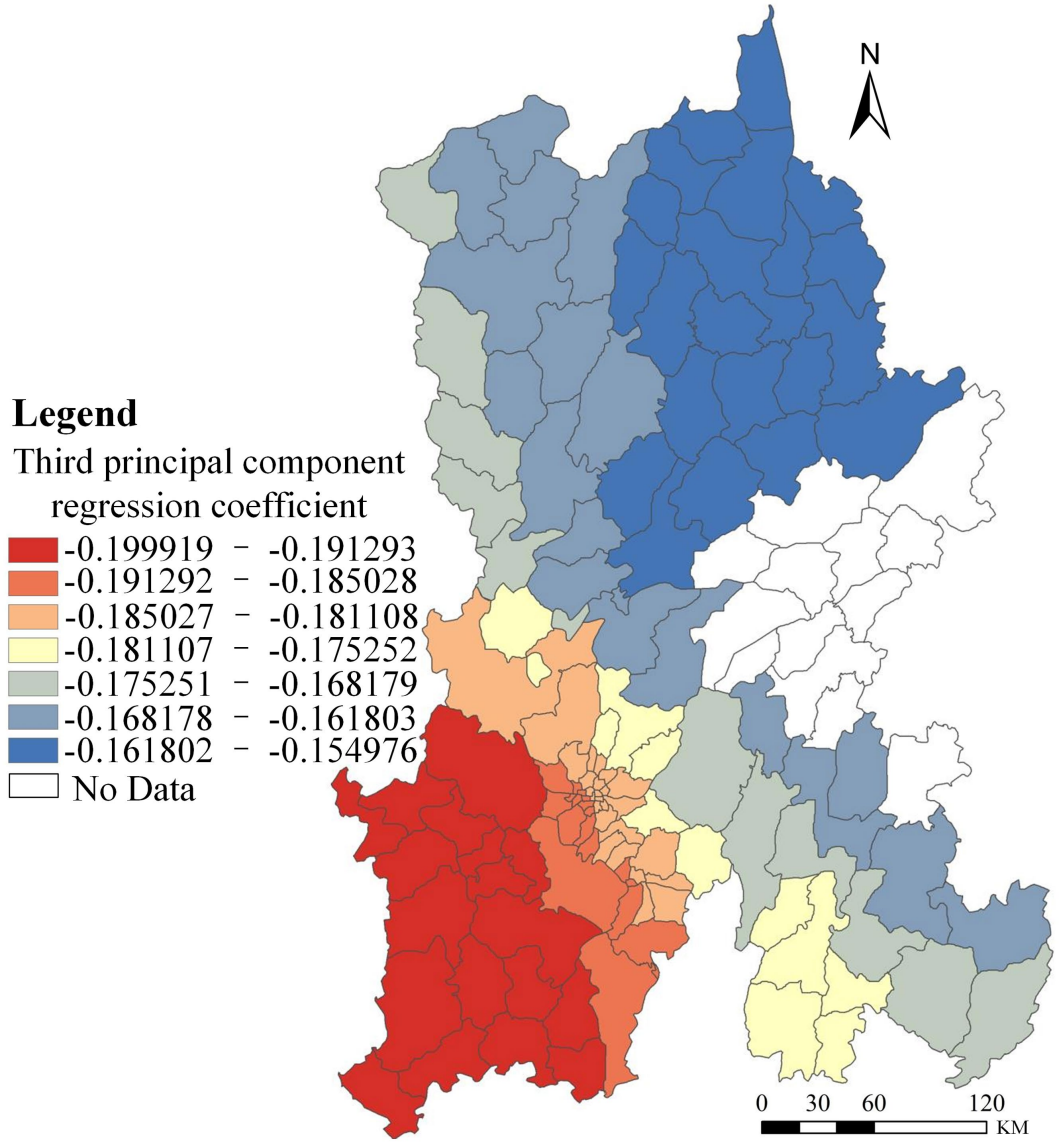
Spatial distribution of regression coefficients for the third principal component PCA-GWR model.

The third principal component primarily reflects healthcare. Its effect on HIV/AIDS prevalence in Kunming was analyzed, and the results are shown in Figure 4.

The effect of healthcare on HIV/AIDS prevalence in Kunming shows a southwest-to-northeast decreasing gradient, with regression coefficients ranging from −0.199919 to −0.154976 (all negative). These results indicate a negative association between healthcare capacity and disease prevalence: areas with stronger healthcare infrastructure tend to have lower AIDS rates. As healthcare infrastructure improves and HIV surveillance and reporting become more standardized, access to facilities increases and counseling and testing services become more widely available.

In Kunming, voluntary counseling and testing (VCT) services are most concentrated in the southern districts—Wuhua, Panlong, Guandu, Xishan, Anning, and Chenggong—where residents enjoy the greatest ease of access to HIV counseling and testing, receive more testing services, and acquire more knowledge of hygienic self-protection. Meanwhile, continual advances in medical technology, nationwide implementation of free antiretroviral therapy (ART), and the broad dissemination of health education have strengthened preventive awareness, thereby facilitating control of the HIV epidemic. In addition, increasing standardization within the medical sector has progressively reduced transfusion-related HIV transmission. It is worth noting, however, that although improvements in medical resources can reduce the risk of HIV infection to some extent, resource-rich areas tend to detect and report more cases in a timely manner, leading to an apparently higher prevalence that reflects enhanced detection rather than a true increase in epidemic risk.

## 5 Discussion

This study applied PCA–GWR model to examine determinants of the HIV/AIDS epidemic in Kunming and underscored the pivotal roles of economic development, educational attainment, and healthcare resources. The results reveal pronounced spatial heterogeneity in the influence of these factors across Kunming.

### 5.1 The spatial impact of economic level

Economic level is widely regarded as a key social determinant of the AIDS epidemic, particularly in low- and middle-income countries (16–18). In Kunming, the effect of economic development on HIV/AIDS prevalence shows clear spatial variation. Areas with lower development—such as Luquan Yi and Miao Autonomous County, Xundian Hui and Yi Autonomous County, Dongchuan District, and Songming County in northern Kunming—exhibit higher prevalence. In these settings, health awareness is relatively weak and limited resources constrain the coverage of prevention and treatment measures. Moreover, low-income populations often experience greater mobility (e.g., labor migration and prolonged separation from family or spouses), which increases the likelihood of engaging in high-risk behaviors such as commercial sex or drug use, thereby elevating HIV transmission risk.

### 5.2 The spatial impact of healthcare level

Healthcare capacity plays a pivotal role in HIV/AIDS prevention and control (18, 19). In Kunming, southern districts with stronger medical and public health conditions (e.g., Wuhua, Panlong, Guandu) show lower HIV/AIDS prevalence, reflecting the significant impact of healthcare resources on epidemic control. Higher levels of care not only facilitate early diagnosis and treatment but also promote earlier intervention by increasing testing and reporting rates. However, in areas with better health infrastructure, the continued expansion of VCT and PITC coverage leads to the identification and reporting of more infections, and these areas may therefore exhibit apparently higher prevalence.

### 5.3 The role of educational level and cultural differences

Educational attainment is strongly associated with HIV/AIDS awareness and preventive behaviors. Findings from the Kunming study indicate that areas with a higher proportion of low-educated populations—such as Xundian Hui and Yi Autonomous County, Luquan Yi and Miao Autonomous County, and Dongchuan District in the northern part of the city—tend to have higher HIV/AIDS prevalence rates. Populations with limited education generally have lower HIV/AIDS knowledge and weaker self-protective behaviors, making them more susceptible to infection and onward transmission. Evidence from low-income, ethnically diverse border regions—such as Sichuan Province and Dehong Prefecture in Yunnan—shows that the rate of late HIV/AIDS diagnosis is highest among illiterate populations (20, 21). Limited knowledge and weak self-protection among low-educated groups substantially increase their risk of infection.

In addition, we found that the unique cultural background of ethnic minority groups is an important factor contributing to the prevalence of HIV/AIDS in multi-ethnic regions, a finding that has also been supported by related studies (22–24). Kunming is a modern, multi-ethnic city that is home to 11 minority groups and is characterized by rich cultural diversity. Ethnic minority populations are more susceptible to HIV/AIDS infection due to limited proficiency in Mandarin, lower levels of education, insufficient awareness of HIV/AIDS, and weaker self-protection consciousness and capacity. Most ethnic minority populations tend to embrace a more naturalistic worldview and hold relatively open attitudes toward sexuality, showing greater tolerance of premarital sex. They generally experience sexual initiation at an earlier age, have multiple sexual partners, and lack both the awareness and access to condom use, leading to a higher prevalence of high-risk sexual behaviors associated with HIV transmission.

### 5.4 General discussion and policy implications

The prevalence of HIV/AIDS is shaped by the complex interplay of multiple contributing factors. The study found that northern areas of Kunming—such as Luquan Yi and Miao Autonomous County, Xundian Hui and Yi Autonomous County, and Dongchuan District—are affected by a combination of economic poverty, limited educational resources, and inadequate healthcare services. In future HIV/AIDS prevention and control efforts, resources should be allocated rationally, and public health infrastructure should be strengthened through increased government investment and the active participation of social organizations. Particular attention should be given to enhancing the supply of HIV/AIDS prevention materials and improving the accessibility of medical services. Second, in the healthcare sector, efforts should be made to strengthen its role in early screening, diagnosis, and standardized treatment of HIV/AIDS. Greater investment from both the government and society is needed to improve the testing and treatment service system, thereby enhancing the accessibility and effectiveness of HIV prevention and control at the primary level. In the field of education, greater emphasis should be placed on disseminating HIV/AIDS-related knowledge, particularly by strengthening sex and health education, to enhance public awareness of HIV/AIDS prevention and control—especially among populations in remote areas and those with lower levels of educational attainment. In the southern areas of Kunming, management should be strengthened to ensure regular follow-up of people living with HIV/AIDS, improve their quality of life, and reduce the risk of HIV transmission from high-risk groups to the general population.

### 5.5 Limitations and future research directions

This study has certain limitations. First, prior research suggests that differences in sex and age may influence infection risk and behavioral patterns (25), yet these demographic characteristics were not incorporated into our analysis. Because all influencing factors were derived from district/county–level data and downscaled to street-level points via spatial interpolation, precise spatial distributions for populations by sex or specific age groups are unavailable. Given the high population mobility in Kunming, existing data cannot accurately characterize the spatial features of stratified populations, thereby limiting in-depth analysis of the potential moderating effects of demographic factors on the HIV/AIDS epidemic.

In addition, the range of variables in this study was limited. Certain potentially important factors closely related to sex and age—such as illicit blood trading and social media use—were not incorporated due to data confidentiality and restricted access. Future work that integrates more detailed census or migrant-population datasets, together with additional variables capturing individual behaviors, would enable a more in-depth exploration of the mechanisms driving the HIV/AIDS epidemic.

Second, 2020 coincided with the COVID-19 pandemic, which disrupted healthcare systems and public health behaviors. On one hand, the pandemic likely reduced the willingness of some populations to seek voluntary testing, leading to undetected infections; on the other hand, the reallocation of attention and resources from HIV programs to COVID-19 response weakened HIV prevention and treatment capacity. Consequently, the 2020 data may underestimate the true level of HIV prevalence.

The integration of PCA and GWR effectively alleviated issues of multicollinearity and spatial non-stationarity, thereby improving the model’s ability to capture spatial heterogeneity in HIV/AIDS prevalence. Because the analysis relies on data aggregated at the administrative-unit level, the findings may still be affected by the modifiable areal unit problem (MAUP). Differences in spatial scale and boundary delineation may exert certain influences on the estimation of model parameters. Although the GWR model can partially mitigate this issue, it cannot completely eliminate its effects. Future research could incorporate higher-resolution raster data or individual-level datasets to further validate the findings and enhance the robustness and generalizability of the results.

## 6 Conclusion

This study examined Kunming using 2020 HIV/AIDS data as the primary dataset. Nineteen potential determinants of HIV/AIDS prevalence were selected, and a PCA–GWR model was applied to analyze their effects. The main findings are summarized below.

1. In 2020, HIV/AIDS prevalence in Kunming was significantly associated with economic development, educational attainment, and healthcare, and the effects of these factors exhibited marked spatial heterogeneity.
2. In northern Kunming—Dongchuan District, Luquan County, Xundian County, and Fumin County—HIV/AIDS prevalence is shaped primarily by the combined effects of economic conditions and educational attainment. Economic development is negatively correlated with prevalence (i.e., lower development corresponds to higher disease burden), whereas the share of low-education populations is positively correlated with prevalence (i.e., larger proportions of less-educated residents are associated with higher prevalence).
3. In southern Kunming—Anning City, Xishan District, Wuhua District, and Panlong District—HIV/AIDS prevalence shows a strong inverse association with healthcare capacity; that is, better healthcare is associated with lower prevalence. However, these areas also tend to have higher detection and reporting rates.
4. For the northern areas of Kunming, where economic development is relatively weak and educational levels are low, financial resources should be appropriately prioritized and health education efforts strengthened at the local level. In economically disadvantaged regions, linking comprehensive HIV/AIDS interventions with social welfare programs—such as integrating free testing and condom provision into basic public health services and offering transport subsidies—can reduce the opportunity costs of accessing care and follow-up. Improving educational attainment in remote areas—particularly in sexual health education—is a key strategy for controlling the spread of HIV/AIDS. Multiple forms of health promotion can be adopted, such as using local ethnic languages and disseminating information through communities and schools, especially among adolescents and young adults, to enhance their awareness of prevention and self-protection.
5. A rational allocation of medical resources combined with regionally differentiated governance is essential for improving the effectiveness of HIV/AIDS prevention and control. For the northern areas of Kunming, where healthcare conditions are relatively poor, improvements in public health infrastructure should be promoted through increased government investment and the active participation of social organizations. Efforts should focus on strengthening the capacity of primary healthcare institutions, particularly by providing greater support for early HIV/AIDS testing, diagnosis, and treatment. In the central and southern areas of Kunming, where medical resources are relatively abundant, relevant authorities should strengthen management, ensure regular follow-up for people living with HIV/AIDS, and improve their quality of life, thereby reducing the transmission of AIDS from high-risk groups to the general population.

## Data Availability

The original contributions presented in the study are included in the article/supplementary material, further inquiries can be directed to the corresponding author.
